# Elevated Lipoprotein(a) Levels are Associated with Arterial Stiffness Measured by Cardio-Ankle Vascular Index in Patients Undergoing Peritoneal Dialysis

**DOI:** 10.31083/j.rcm2411322

**Published:** 2023-11-23

**Authors:** Po-Yu Huang, Bang-Gee Hsu, Huei-Jhen Lin, Yu-Li Lin, Chih-Hsien Wang, Jen-Pi Tsai

**Affiliations:** ^1^Division of Nephrology, Dalin Tzu Chi Hospital, Buddhist Tzu Chi Medical Foundation, 62247 Chiayi, Taiwan; ^2^School of Medicine, Tzu Chi University, 97004 Hualien, Taiwan; ^3^Division of Nephrology, Hualien Tzu Chi Hospital, Buddhist Tzu Chi Medical Foundation, 97004 Hualien, Taiwan

**Keywords:** cardio-ankle vascular index, arterial stiffness, lipoprotein(a), peritoneal dialysis

## Abstract

**Background:**

Arterial stiffness (AS) can be used to predict future cardiovascular diseases. High lipoprotein(a) (Lp(a)) levels were independently correlated with cardiovascular (CV) morbidity and death in patients with chronic renal insufficiency. The cardio-ankle vascular index (CAVI) is a useful biomarker of arteriosclerotic disorders and has a close relationship with a variety of CV events. This study aimed to investigate the correlation between serum Lp(a) levels and AS in patients on peritoneal dialysis (PD) using the CAVI.

**Methods:**

A total of 86 adult patients who were on regular PD for at least 3 months were recruited in this study. The CAVI values were determined using the waveform device (VaSera VS-1000). A CAVI value of ≥9.0 on either side was defined as high. Serum Lp(a) levels were measured by an enzyme-linked immunosorbent assay.

**Results:**

Among these participants, 35 of 86 (40.7%) belonged to the high CAVI group. In contrast to those with a normal CAVI, PD recipients in the high CAVI group had higher serum levels of total cholesterol (*p* = 0.003), triglycerides (*p* = 0.044), C-reactive protein (*p* < 0.001), and Lp(a) (*p* < 0.001), whereas their albumin levels were significantly lower (*p* = 0.026). Based on multivariable logistic regression analysis, serum Lp(a) (odds ratio [OR] 1.025, 95% confidence interval [CI] 1.010–1.040, *p* = 0.001), total cholesterol (OR 1.042, 95% CI 1.005–1.081, *p* = 0.027), and C-reactive protein (each increase 0.1 mg/dL, OR 1.217, 95% CI 1.008–1.469, *p* = 0.041) levels were found as the parameters that could independently predict AS in patients on PD. Further, using Spearman’s correlation analysis, both the left and right CAVIs revealed a significantly positive correlation with log-transformed Lp(a) levels (*r* = 0.588, *p* < 0.001; *r* = 0.639, *p* < 0.001, respectively).

**Conclusions:**

Serum Lp(a) levels were postulated to participate in the pathogenic processes of AS in adult patients undergoing PD.

## 1. Introduction

Cardiovascular (CV) diseases contribute to a significant proportion of comorbidity conditions and mortality in patients with end-stage kidney disease (ESKD) [1]. Risk factors for CV diseases in patients undergoing regular peritoneal dialysis (PD) are classified as traditional (hypertension (HTN), diabetes mellitus (DM), dyslipidemia, age, sex, and family history), nontraditional (inflammation, oxidative stress, endothelial dysfunction, and protein carbamylation), and uremia-specific (uremic toxins, volume overload, hyperparathyroidism, hyperphosphatemia, and vascular calcification) factors [2]. Increased arterial stiffness (AS) is a critical estimator of CV events, CV mortality, and death in patients with ESKD [3, 4]. Pathogenetic factors underlying arterial stiffening included a change in the elastin-to-collagen ratio, altered lipid metabolism, oxidative stress, genetic mutations, and epigenetic regulation [5]. Furthermore, a close association was reported between AS and atherosclerosis [6, 7].

The cardio-ankle vascular index (CAVI) is a noninvasive parameter of global AS from the aorta to the tibial arteries. Compared with the carotid–femoral pulse wave velocity (PWV), the CAVI was considered to be less operator dependent and not influenced by blood pressure during the measurement [8, 9]. The CAVI was reported as being positively associated with the left atrial chamber size, indices of left ventricular diastolic dysfunction, such as the E/A ratio, and the left ventricular mass index [10]. In a small cross-sectional study in Taiwan, patients receiving PD regularly demonstrated a high prevalence of AS, when measured by the CAVI [11]. Compared with the general population, patients on hemodialysis have a significantly higher CAVI [12], which can be used to predict all-cause deaths in individuals on regular hemodialysis therapy [13].

Lipoprotein(a) (Lp(a)), which is produced primarily in the hepatic parenchyma, comprises a low-density lipoprotein core complexed with glycoprotein apo(a). High plasma Lp(a) concentrations indicate an increased risk for CV diseases [14, 15]. The proatherogenic, prothrombotic, and proinflammatory effects of Lp(a) accelerate atherosclerotic plaque formation [15]. Furthermore, the serum Lp(a) concentrations increase significantly as the glomerular filtration rate declines. Patients on chronic dialysis therapy have higher Lp(a) levels than those without kidney failure [16]. Histologically, high plasma Lp(a) concentrations were found to be positively correlated with arterial Lp(a) content and atherosclerosis progression in patients with ESKD [17]. In a cohort study that focused on PD recipients, the upper tertile of Lp(a) levels had the highest CV mortality rate but not all-cause mortality [18].

Thus, this study aimed to assess the association between the serum Lp(a) levels and AS in ESKD individuals on regular PD using the CAVI.

## 2. Materials and Methods

### 2.1 Study Participants

This cross-sectional study was performed in a medical center in Hualian, Taiwan, and had been reviewed and approved by the Institutional Review Board of the Protection of Human Subjects (IRB108-219-A). From March 2020 to October 2021, a total of 86 participants were included who had been on PD for at least 3 months. Among these participants, 32 received continuous ambulatory PD, whereas the remaining 54 were on automated PD. Patients who were not able to offer informed consent or had an underlying malignancy, ongoing infection, heart failure, acute coronary syndrome, stroke, or limb amputation were excluded from the study.

Data on clearance and dialysis adequacy, including weekly and peritoneal fractional clearance index for urea (Kt/V), and total and peritoneal creatinine clearance were extracted from medical records. After the participants had rested for 10 min, the trained staff measured the systolic blood pressure (SBP) and diastolic blood pressure (DBP) three times using standard mercury sphygmomanometers with proper cuff sizes; the interval between each measurement was 5 minutes. HTN was defined as an SBP of 140 mmHg or higher, a DBP of 90 mmHg or higher, and/or the regular usage of antihypertensives over the past 2 weeks. Patients were diagnosed as having DM if the fasting plasma glucose level was ≥126 mg/dL or if they were taking regular oral antidiabetic drugs and/or insulin injections. Coronary artery disease (CAD) was defined by coronary angiography of more than 50% narrowing in any one of three epicardial coronary arteries.

### 2.2 Anthropometric Analyses

With each patient wearing airy clothes and standing without wearing shoes, their weights were measured to the closest half kilogram and heights to the nearest half centimeter. Body mass index (BMI) values were derived from weight/${\mathrm{height}}^{2}$ (kg/$m^{2}$).

### 2.3 Biochemical Investigations

A fasting (at least 8 h) blood specimen of 5 mL was obtained from each study participant before peritoneal dialysate exchange in the day. We obtained blood samples of approximately 0.5 mL to determine the blood cell count for each patient (Sysmex SP-1000i, Sysmex American, Mundelein, IL, USA). The remaining blood volumes were immediately centrifuged at 3000 ×g for 10 minutes. Within one hour of collection, the serum was set aside at 4 °C for further biochemical analyses.

Albumin, total cholesterol, triglycerides, fasting glucose, blood urea nitrogen, creatinine, total calcium, phosphorus, and C-reactive protein (CRP) serum concentrations were determined by an autoanalyzer (Siemens Advia 1800, Siemens Healthcare GmbH, Henkestr, Germany). Intact parathyroid hormone (iPTH) (Catalog no. NM59041; IBL International, Hamburg, Germany) and Lp(a) (Catalog no. ab212165; Abcam, Cambridge, MA, USA) levels were quantified using enzyme-linked immunosorbent assays.

### 2.4 Measurement of Carotid Ankle Vascular Index

CAVI values were estimated using the waveform device (VaSera VS-1000, Fukuda Denshi Co. Ltd., Tokyo, Japan), in accordance with previous recommendations [9, 19]. After the participants were kept in the supine position for 10 minutes, with continuous monitoring of the electrocardiogram and phonocardiogram, their blood pressure was measured in the arms and ankles. Then, the PWV and CAVI were calculated automatically. Patients with CAVI values of ≥9.0 belonged to the high CAVI group, whereas those with CAVI values of less than 9.0 were included in the normal CAVI group [20].

### 2.5 Statistical Analyses

Variables that were continuous were determined as normally distributed by the Kolmogorov–Smirnov test. Normally distributed continuous variables were represented as means ± standard deviations; for comparison between the two groups, the two-tailed independent Student’s *t*-test was employed. Variables with nonnormal distributions were displayed as median and interquartile ranges, with between-group comparisons using the Mann–Whitney U test. Data without normal distribution were logarithmically transformed before further linear regression analysis. Qualitative variables were shown as numbers (percentages) and analyzed by the χ^2^ test. Based on the results of multivariable logistic regression analysis, we could identify the potential risk factors for AS. To investigate the relationship between the clinical parameters and CAVI (left and right) and Lp(a), Spearman’s rank correlation coefficient was used. These data were analyzed using IBM SPSS Statistics for Windows version 19.0 (IBM Corp., Armonk, NY, USA). A *p*-value less than 0.05 was regarded as statistically significant.

## 3. Results

The baseline parameters of the 86 patients undergoing PD are shown in Table 1. Major chronic medical illnesses included DM (n = 39; 45.3%) and HTN (n = 68; 79.1%). A total of 35 patients exhibited a high CAVI. Compared with the group with a normal CAVI, the patients with a higher CAVI had lower serum albumin concentrations (*p* = 0.026) and higher total cholesterol (*p* = 0.003), triglyceride (*p* = 0.044), CRP (*p* < 0.001), and Lp(a) (*p* < 0.001) levels. Blood hemoglobin and serum levels of fasting glucose, blood urea nitrogen, creatinine, total calcium, phosphorus, iPTH, age, sex, BMI, blood pressure, total and dialysate clearance of solutes, smoking status, anti-DM drugs used, anti-HTN drugs used, and proportions of patients with underlying DM, HTN, and CAD did not differ significantly between the groups.

**Table 1.** **Clinical variables for peritoneal dialysis patients with normal or high cardio-ankle vascular index scores**.

Characteristic	All participants (n = 86)	Normal CAVI group (n = 51)	High CAVI group (n = 35)	*p*-value
Age (years)	58.56 ± 14.33	57.00 ± 15.65	60.83 ± 12.02	0.226
Peritoneal dialysis vintage (months)	48.54 (21.00–82.32)	54.24 (21.00–88.92)	34.44 (21.00–69.12)	0.257
Height (cm)	159.83 ± 8.63	158.33 ± 8.63	162.00 ± 8.25	0.052
Body weight (kg)	64.28 ± 13.41	64.62 ± 14.03	63.80 ± 12.64	0.783
Body mass index (kg/$m^{2}$)	25.06 ± 4.32	25.65 ± 4.53	24.20 ± 3.90	0.129
Left CAVI	7.79 ± 2.52	6.34 ± 1.77	9.91 ± 1.87	<0.001*
Right CAVI	7.67 ± 2.36	6.25 ± 1.77	9.74 ± 1.37	<0.001*
Systolic blood pressure (mmHg)	145.77 ± 18.82	145.73 ± 19.65	145.83 ± 17.81	0.980
Diastolic blood pressure (mmHg)	83.4 ± 10.42	83.96 ± 10.06	83.43 ± 11.05	0.817
Hemoglobin (gm/dL)	9.62 ± 1.43	9.57 ± 1.50	9.70 ± 1.33	0.678
Albumin (g/dL)	3.54 ± 0.35	3.61 ± 0.33	3.44 ± 0.35	0.026*
Total cholesterol (mg/dL)	162.72 ± 43.17	151.29 ± 40.59	179.37 ± 41.89	0.003*
Triglyceride (mg/dL)	139.13 ± 77.52	125.20 ± 63.76	159.43 ± 91.27	0.044*
Fasting glucose (mg/dL)	104.00 (91.00–129.25)	105.00 (91.00–123.00)	103.00 (91.00–137.00)	0.778
Blood urea nitrogen (mg/dL)	61.53 ± 22.40	60.49 ± 23.99	63.06 ± 20.08	0.604
Creatinine (mg/dL)	10.28 ± 3.31	10.46 ± 3.44	10.02 ± 3.16	0.545
Total calcium (mg/dL)	9.62 ± 0.62	9.60 ± 0.63	9.66 ± 0.61	0.645
Phosphorus (mg/dL)	5.22 ± 1.33	5.39 ± 1.36	4.97 ± 1.25	0.153
Intact parathyroid hormone (pg/mL)	184.35 (78.33–446.63)	190.00 (89.10–470.10)	150.90 (54.00–338.10)	0.345
C-reactive protein (mg/dL)	0.35 (0.14–0.99)	0.18 (0.11–0.60)	0.60 (0.31–1.11)	<0.001*
Lipoprotein(a) (mg/L)	403.56 ± 187.38	278.58 ± 95.13	585.67 ± 129.99	<0.001*
Weekly Kt/V	1.97 (1.71–2.18)	1.95 (1.66–2.12)	2.00 (1.75–2.34)	0.166
Peritoneal Kt/V	1.78 (1.51–1.98)	1.77 (1.48–1.99)	1.78 (1.54–1.98)	0.492
Total clearance of creatinine (L/week)	58.64 ± 16.67	60.31 ± 15.31	56.22 ± 18.44	0.266
Peritoneal clearance of creatinine (L/week)	47.52 ± 12.30	49.47 ± 12.59	44.68 ± 11.45	0.075
Female, n (%)	50 (58.1)	31 (60.8)	19 (54.3)	0.548
Diabetes, n (%)	39 (45.3)	21 (41.2)	18 (51.4)	0.348
Hypertension, n (%)	68 (79.1)	39 (76.5)	29 (82.9)	0.474
CAPD model, n (%)	32 (37.2)	19 (37.3)	13 (37.1)	0.992
Smoking, n (%)	9 (10.5)	5 (9.8)	4 (11.4)	0.809
Coronary artery disease, n (%)	19 (22.1)	10 (19.6)	9 (25.7)	0.502
Calcium channel blockers, n (%)	45 (52.3)	27 (52.9)	18 (51.4)	0.891
Angiotensin receptor blockers, n (%)	46 (53.5)	28 (54.9)	18 (51.4)	0.753
Alpha-adrenergic blockers, n (%)	10 (11.6)	7 (13.7)	3 (8.6)	0.467
Beta-adrenergic blockers, n (%)	32 (37.2)	19 (37.3)	13 (37.1)	0.992
Aspirin, n (%)	10 (11.6)	5 (9.8)	5 (14.3)	0.527
Statins, n (%)	20 (23.3)	11 (21.6)	9 (25.7)	0.657
Fibrates, n (%)	6 (7.0)	4 (7.8)	2 (5.7)	0.705
Insulin, n (%)	12 (14.0)	7 (13.7)	5 (14.3)	0.947
Sulfonylureas, n (%)	7 (8.1)	3 (5.9)	4 (11.4)	0.355
Repaglinide, n (%)	11 (12.8)	6 (11.8)	5 (14.3)	0.731
Dipeptidyl peptidase-4 inhibitors, n (%)	25 (29.1)	16 (31.4)	9 (25.7)	0.570

The continuous variable results are presented as mean ± standard deviation after being analyzed by Student’s *t*-test; variables that were not normally distributed are illustrated as median and interquartile range after analysis by the Mann-Whitney U test; values that are qualitative in nature are presented as number (%) and were assessed using the Chi-squared test. CAVI, cardio-ankle vascular index; weekly Kt/V, weekly fractional clearance index for urea; CAPD, continuous ambulatory peritoneal dialysis. *p values of <0.05 were considered statistically significant.

Multivariate logistic regression analysis was performed to identify the aspects independently correlated with AS and adjusted for albumin, total cholesterol, triglyceride, CRP, Lp(a), age, and gender. Lp(a) [odds ratio (OR) 1.025; 95% confidence interval (CI) 1.010–1.040; *p* = 0.001], total cholesterol (OR 1.042; 95% CI 1.005–1.081; *p* = 0.027), and CRP (each increase 0.1 mg/dL, OR 1.217; 95% CI 1.008–1.469; *p* = 0.041) were the independent risk factors for AS development among the study patients (Table 2).

**Table 2.** **Multivariable logistic regression analysis of the factors correlated to cardio-ankle vascular index in patients undergoing peritoneal dialysis**.

Variables	Odds ratio	95% Confidence interval	*p*-value
Lipoprotein(a), 1 mg/L	1.025	1.010–1.040	0.001*
Total cholesterol, 1 mg/dL	1.042	1.005–1.081	0.027*
C-reactive protein, 0.1 mg/dL	1.217	1.008–1.469	0.041*
Albumin, 1 g/dL	0.068	0.001–3.949	0.195
Triglyceride, 1 mg/dL	1.004	0.988–1.020	0.654
Age, 1 year	1.019	0.926–1.122	0.699
Female	0.143	0.007–2.866	0.203

Analysis data were determined using the multivariable logistic regression analysis (adopted factors: albumin, total cholesterol, triglyceride, C-reactive protein, lipoprotein (a), age, and gender). *Values of *p <* 0.05 were considered statistically significant.

Table 3 represents the link between the baseline parameters and CAVI (left and right) as well as serum log-transformed Lp(a) (log-Lp(a)) levels by Spearman’s correlation analysis. Both the left and right CAVIs showed a significant and positive correlation with serum log-Lp(a) levels (*r* = 0.588, *p* < 0.001; *r* = 0.639, *p* < 0.001, respectively). Moreover, serum log-Lp(a) levels were positively associated with serum total cholesterol (*r* = 0.218, *p* = 0.043) and log-CRP (*r* = 0.270, *p* = 0.012) levels in addition to BMI (*r* = –0.243, *p* = 0.024). Both the left and right CAVIs had a significant positive correlation with serum log-CRP levels (*r* = 0.349, *p* = 0.001; *r* = 0.373, *p* < 0.001, respectively) and a negative correlation with serum albumin levels (*r* = –0.255, *p* = 0.018; *r* = –0.223, *p* = 0.039, respectively).

**Table 3.** **Spearman correlation coefficients between left CAVI, right CAVI, lipoprotein(a), and clinical characteristics in patients on regular peritoneal dialysis therapy**.

Variables	Left CAVI		Right CAVI		Log-Lp(a) (mg/L)	
	Spearman’s coefficient of correlation	*p*-value	Spearman’s coefficient of correlation	*p-*value	Spearman’s coefficient of correlation	*p*-value
Age (years)	0.109	0.320	0.182	0.093	0.163	0.133
Body mass index (kg/$m^{2}$)	–0.147	0.176	–0.167	0.123	–0.243	0.024*
Left CAVI	**—**	**—**	0.824	<0.001*	0.588	<0.001*
Right CAVI	0.824	<0.001*	**—**	**—**	0.639	<0.001*
Log-Lp(a) (mg/L)	0.588	<0.001*	0.639	<0.001*	**—**	**—**
Log-PD vintage (months)	0.050	0.645	0.072	0.507	–0.004	0.971
SBP (mmHg)	–0.018	0.873	–0.033	0.763	–0.083	0.449
DBP (mmHg)	–0.127	0.244	–0.152	0.163	–0.105	0.335
Hemoglobin (gm/dL)	0.039	0.724	0.073	0.504	0.065	0.554
Total cholesterol (mg/dL)	0.143	0.190	0.147	0.177	0.218	0.043*
Triglyceride (mg/dL)	0.139	0.201	0.162	0.136	0.181	0.096
BUN (mg/dL)	–0.179	0.099	–0.078	0.474	–0.068	0.533
Creatinine (mg/dL)	–0.139	0.200	–0.098	0.372	0.038	0.727
Log-glucose (mg/dL)	0.023	0.833	0.012	0.909	0.020	0.857
Albumin (g/dL)	–0.255	0.018*	–0.223	0.039*	–0.202	0.062
Total calcium (mg/dL)	0.070	0.522	0.185	0.089	0.186	0.086
Phosphorus (mg/dL)	–0.158	0.145	–0.210	0.052	–0.159	0.144
Log-iPTH (pg/mL)	–0.119	0.276	–0.111	0.311	–0.057	0.601
Log-CRP (mg/dL)	0.349	0.001*	0.373	<0.001*	0.270	0.012*

Data for PD vintage, glucose, iPTH, CRP, and Lp(a) levels revealed a skewed distribution and were, therefore, log-transformed before any subsequent analyses. Data analysis was performed using Spearman’s correlation analysis method. CAVI, cardio-ankle vascular index; Lp(a), lipoprotein(a); PD, peritoneal dialysis; SBP, systolic blood pressure; DBP, diastolic blood pressure; BUN, blood urea nitrogen; iPTH, intact parathyroid hormone; CRP, C-reactive protein. *Values of *p <* 0.05 were considered statistically significant.

## 4. Discussion

The most important findings in the study were that the Lp(a), total cholesterol, and CRP serum concentrations were independently related to AS in patients undergoing chronic PD therapy. Both the left and right CAVIs are also positively associated with the serum log-Lp(a) levels. It is possible to use Lp(a) to predict adverse CV outcomes in these patients.

The pathophysiology of the Lp(a) contribution to the atherosclerotic process is complex. Several proposed mechanisms explain the contributing role of Lp(a) in atherogenesis, including direct Lp(a) deposition in arterial walls, increased foam cell transformation, and induction of endothelial dysfunction [15]. The apolipoprotein(a) (apo(a)) domain of Lp(a) shows homology to plasminogen; hence, Lp(a) interferes with the ability of plasminogen to facilitate fibrinolysis and promote thrombi formation [15, 21]. High serum Lp(a) levels were positively associated with platelet aggregation, which is an alternative explanation of the thrombogenic effect of Lp(a) [22, 23]. Lp(a) accelerates coagulation by promoting tissue factor expression and inhibiting the activity of the tissue factor pathway inhibitors [24, 25, 26]. Furthermore, Lp(a) has a proinflammatory role in atherosclerosis. Lp(a) and associated oxidized phospholipids upregulate proinflammatory cytokines, such as interleukin-6 (IL-6), IL-1β, and tumor necrosis factor-α, increase the expression of adhesion molecules, and induce monocyte chemotaxis and migration [26, 27]. In summary, Lp(a) has several roles in atherosclerosis, including direct deposition in arterial walls, increased foam cell transformation, induction of endothelial dysfunction, and promotion of thrombi formation. It also promotes coagulation by promoting tissue factor expression and inhibiting the activity of the tissue factor pathway inhibitors. In this study, the serum log-Lp(a) levels in PD recipients were also positively associated with log-CRP levels.

Both Lp(a) and AS are important biomarkers of atherosclerosis and CV diseases. The association between Lp(a) and AS has been extensively studied. In a cross-sectional study that recruited hypertensive patients, AS was determined by measuring the carotid–femoral PWV and calculating the brachial augmentation index. The study concluded that logarithmically transformed Lp(a) and CRP were independently associated with an increased index but not with carotid–femoral PWV [28]. Furthermore, in older patients with DM, the positive correlation between serum Lp(a) and AS, determined by measuring the aortic PWV, was independent of age, sex, glycemic control, and the use of lipid-lowering medications [29]. In another small study, which included female participants, among the participants with hypertension, only oxidized Lp(a) concentrations, and not Lp(a) levels, were positively and independently correlated with the CAVI [30]. However, whether oxidized Lp(a) has a better predictive role in AS or adverse CV outcomes is still under investigation [31]. Moreover, the efficacy of Lp(a)-lowering therapy on CV endpoints is still not well established [32].

Cholesterol is an important constituent in atherosclerotic plaques; hyperlipidemia is a traditional CV risk factor. Establishing a positive association between hypercholesterolemia and AS was based on patients with familial hypercholesterolemia [33]. In a large prospective community-based study, total cholesterol concentrations were positively correlated with the brachial–ankle PWV and cholesterol could mediate hypertension through AS [34]. However, in a cohort study of older patients with hypertension, the total cholesterol levels did not correlate with large AS, when measured by aortic distensibility, increased index, and systemic arterial compliance [35]. On the contrary, serum triglyceride levels were significantly higher in the high CAVI group; however, triglycerides failed to independently predict AS in PD recipients. In a prospective community-based study, triglyceride levels were associated with AS independently of multiple CV risk factors and statin therapy [36].

The contributing role of malnutrition and inflammation in atherosclerosis is well-established [37]. The pathophysiology is characterized by the expression of adhesion molecules in endothelial cells, monocyte migration and penetration into vessel walls, macrophage transformation into foam cells, generation of proinflammatory cytokines and reactive oxygen species, activation of the coagulation cascade with a tendency of thrombus formation, and further activation of T lymphocytes [38, 39]. Moreover, CRP is not only a marker of inflammation but also participates in the progression of atherosclerosis and intravascular thrombosis [40]. Among PD recipients, higher CRP levels are an independent risk factor for the development of CV diseases, while high CRP levels were an independent predictor of the increased incidence of CV events [41, 42]. Several studies have found an association between CRP concentrations and AS, with the study participants including healthy and older individuals [43, 44]. PD recipients with protein-energy wasting have a reverse correlation between albumin levels and CRP, and IL-6 levels [45]. In addition, the aortic PWV is inversely correlated with albumin in PD recipients [46]. In this study, the left and right CAVIs were significantly and positively linked to the serum log-CRP levels and negatively linked to the serum albumin levels. Furthermore, serum CRP levels in patients undergoing chronic PD therapy, after adjusting for albumin, total cholesterol, triglycerides, and Lp(a), were independently associated with AS.

Previous investigations indicated that low Lp(a) levels could predict new-onset diabetes when the serum Lp(a) was inversely proportional to insulin concentrations and degree of insulin resistance [47, 48]. However, in the present study, we did not find any significant correlation between fasting plasma glucose or underlying DM and Lp(a) serum levels among patients undergoing PD.

As seen in Table 3, the serum calcium concentrations were slightly positively correlated with the right CAVI, whereas phosphorus concentrations were slightly negatively correlated, although neither reached statistical significance. There was no association between the iPTH and CAVI. Vascular calcifications are a critical component of chronic kidney disease mineral and bone disorder (CKD-MBD). Previous studies revealed that higher serum calcium levels, as a higher iPTH, were positively linked to markers of vascular stiffening, such as PWV [49]. In another study on PD patients, hyperphosphatemia was found to independently predict the deterioration of coronary artery calcifications [50]. The mixed results explained the complexity of CKD-MBD; various factors such as fibroblast growth factor-23, vitamin D, and other promoters or inhibitors of vascular calcifications could also contribute to the process.

There are a few limitations in the current investigation. Firstly, there was a limited number of samples and the study was conducted in a single center. Secondly, the cross-sectional design limited the ability of the study to determine causal relationships between clinical variables and AS. Thirdly, we did not measure the serum oxidized Lp(a) concentrations in the study participants; however, whether oxidized Lp(a) has a better predictive value for AS than conventional Lp(a) needs further investigation.

## 5. Conclusions

In this study on patients with ESKD undergoing maintenance PD, along with serum total cholesterol and CRP levels, the most significant clinical implication was that serum Lp(a) levels were positively correlated to the CAVI and can be used as a potential novel biomarker of AS. Currently, whether higher Lp(a) concentrations adversely affect CV morbidity and long-term survival among these patients is unknown. Thus, further prospective studies with a longitudinal design are warranted to determine the causal role of Lp(a) on clinical outcome parameters.

## Data Availability

The data presented in this study are available on request from the corresponding author.
